# Supplementary material for: Cutting consumption without diluting the experience: Preferences for different tactics for reducing alcohol consumption among increasing-and-higher-risk drinkers based on drinking context
Source: PLOS Digit Health. 2024 Aug 21;3(8):e0000523. doi: 10.1371/journal.pdig.0000523 (PMC11338454; doi:10.1371/journal.pdig.0000523)
Supplement: S1 Appendix — (DOCX) [file pdig.0000523.s001.docx]

**S1 Appendix:** Topic Guide

| Phase |  | Questions |
| --- | --- | --- |
| **Phase 1-**  **Introduction**  **(10 minutes)** | Introduce the facilitators (MO and TO/CL) and talk about the type of research interests each has.  Give introduction to Drink Less app and explain the goal to provide intervention content which is tailored to different types of drinking occasions:  “*The goal of the focus group is to get your feedback on some changes we are making to an existing alcohol reduction app, Drink Less. As part of this you will not be expected to share any personal information if you do not want to. If it is helpful for you when answering questions you can answer in third person. For example, if we ask you if you think a piece of advice is helpful you could answer by saying “this might help some people…” rather than talking about yourself if you prefer*.”  Explain presence of the audio recorders.  Go over consent and what this means. Ask participants to reaffirm consent.  Go over focus group “ground rules” and confidentiality:  “*All information collected today will be confidential and no one’s name will be disclosed or linked to any quotes in the final report. As this is a group discussion, please respect the privacy of your fellow participants and not share the contents of this discussion outside of this room.*  *We all have different views and experiences and its ok if you don’t always agree with other people in the group. I am keen to hear from everybody so please be respectful of each other’s opinions and encourage everybody to share. We have a limited amount of time so please do not be offended if I stop you to redirect the conversation slightly or to bring someone else in. I will start by asking a few questions but really I want most of the session to be a conversation between you which I will occasionally direct towards a certain topic. I hope this encourages you all to speak openly and thank you all for your participation*”. | |
| **Phase 2 – Introduction to the Drink Less App. (10 minutes)** | Opening Question | Can we go around the room and introduce ourselves, giving your first name. I would also like you to tell us what is your favourite app and why?  Prompts:  What do you like about it?  Is there anything you didn’t like about it? |
| **Phase 3 – Insights Component (25 minutes)** | Introduction | Explaining the “Insights” component in more detail. Explain that this is a starting point, that we know they aren’t perfect yet and we are looking for help in making them better. |
|  | Key Questions | 1.What do you think of the following wording?  “When you drink more units than you want to you tend to be drinking *occasion X*  When you drink on more days than you want to you tend to be drinking *occasion X*  When you spend more than you wanted to you tend to be drinking *occasion X*  When you have more calories than you wanted to you tend to be drinking *occasion X”*  2. How would you feel about receiving this feedback?  3. If you were going to say this in your own words how would you write it?  4. Would you find this clear or helpful?  5. Would anything about this annoy you? |
| **Phase 4 – Action Planning Component (45 minutes)** | Introduction | Explaining the “Action Planning” component in more detail. Explain that this is a starting point, that we know they aren’t perfect yet and we are looking for help in making them better. |
|  | Card Sorting Task | Group 1: Alone at home and Pub with friends  Group 2: Pub with friends and Home with partner/family  Group 3: Home with partner/family and Meal Out  Group 4: Meal Out and Alone at Home  *Context 1*  We have developed advice which would be helpful for people drinking in “Context X”.  Please could you as a group place each card on this line ranging from ‘very helpful’ at one end to ‘not at all helpful’ at the other.  After task:   1. Can you choose some of the advice you did find helpful and explain why? 2. Can you choose a couple of the cards you thought were less helpful and explain why? 3. Why were the cards in the middle ranked as belonging there? 4. Would any of the cards be annoying or would they aggravate you? 5. Did anyone feel differently from the group or would have put them in a different order? 6. Are there any which did not make sense to you?   Prompts   1. How would receiving that advice make you feel? 2. Do you think you would follow that advice? 3. Does anyone disagree?   *Context 2*  We have developed advice which would be helpful for people drinking in “context X”.  Please could you as a group place each card on this line ranging from ‘very helpful’ at one end to ‘not at all helpful’ at the other.  After task:   1. Can you choose some of the advice you did find helpful and explain why? 2. Can you choose a couple of the cards you thought were less helpful and explain why? 3. Why were the cards in the middle ranked as belonging there? 4. Would any of the cards be annoying or would they aggravate you? 5. Did anyone feel differently from the group or would have put them in a different order? 6. Are there any which did not make sense to you?   Prompts   1. How would receiving that advice make you feel? 2. Do you think you would follow that advice? 3. Does anyone disagree? |
| **Phase 5 – Debrief (5 minutes)** | Ending questions | 1. Does anyone have anything else they would like to share? 2. Does anyone have any questions? Clarify any misconceptions (if relevant).   Give out any remaining refreshments.  Advise participants that they will be emailed their compensation.  Thank them again for participating. |
