# Supplementary material for: Cutting consumption without diluting the experience: Preferences for different tactics for reducing alcohol consumption among increasing-and-higher-risk drinkers based on drinking context
Source: PLOS Digit Health. 2024 Aug 21;3(8):e0000523. doi: 10.1371/journal.pdig.0000523 (PMC11338454; doi:10.1371/journal.pdig.0000523)
Supplement: S2 Appendix — (DOCX) [file pdig.0000523.s002.docx]

**S2 Appendix: Reflexivity**

Reflexivity

*Personal Characteristics of Interviewers*: There were three facilitators. MO is a female senior research fellow at UCL and has a PhD in Health Psychology. She is a mixed-methods researcher and has previously conducted semi-structured interviews and thematic analysis. MO has attended training courses for facilitating focus groups.

TO is a female PhD student at UCL with an MSc in Public Health and Health Promotion. She is a mixed-methods researcher with previous experience conducting and analysing data from focus groups and has attended training courses in interviewing.

CL is a female PhD student at UCL with an MSc in Health Psychology. She primarily does quantitative research. Before this study, CL underwent additional training in qualitative research, focus group facilitation, and qualitative analysis.

*Relationship with Participants*: Participants had not met the researchers before the focus groups but had exchanged emails regarding logistics of focus group. Background on the research focus of the two researchers was included at the start of the focus groups and the goals of the specific research was explained to participants at the start of the focus group.

*Data Collection*: That we are developing an intervention to encourage alcohol reduction may indicate to participants that we have opinions on alcohol consumption and drinking practices. We are aware that this could create a perceived imbalance of power. Participants might feel pressured to hide how much or the ways in which they drink, or to present a version of themselves that is more concerned about drinking less. We aimed to create a non-judgemental and safe environment in which participants felt able to express their views honestly and openly. To achieve this, the lead facilitator MO shared that they drink alcohol, sometimes more than they intend to. Participants were also told that they could share views in the third person if they preferred, so rather than talking about what they might find helpful, focusing on ‘what could be helpful for other people’. The difference in modalities did seem to have some impact on the dynamics of the focus group. It seemed easier in person for participants to signal to others that they had something to say and so a space was left for their insights. This was a little clumsier online with participants occasionally starting to talk at the same time, this may have led participants to be less willing to share views. However, having online focus did seem to be more inclusive and captured voices that otherwise may not have been heard.

*Analysis:* MO undertook an initial analysis. One of MO’s primary research interests is the development of context-specific interventions for alcohol reduction and so a potential bias may be in interpreting transcripts to support the idea that different strategies may be more or less helpful for different contexts. Whilst complete detachment on the part of the researcher in relation to data collection and analysis is unattainable, steps were taken to minimise bias. This included a data driven, systematic analysis strategy and verification with co-facilitators (TO and CL work on novel interventions for smoking cessation which do not focus on alcohol or context) at an early stage in analysis and the full research team (with a diverse range of research interests) at a later stage.

Changes to pre-registration

We had initially planned to examine drinking in the pub alone, rather than drinking at home with partner or family. But participants seemed to be drawing distinctions based on drinking alone versus drinking with friends which mirrored the in-home/out-of-home contexts and so introducing a context where someone was drinking at home with others, but in a more habitual rather than social context seemed an interesting contrast. Furthermore, very few potential participants reported drinking alone in the pub whereas many reported drinking at home with partner or family, so this seemed to be more within the experiences of participants.
