# Supplementary material for: Cutting consumption without diluting the experience: Preferences for different tactics for reducing alcohol consumption among increasing-and-higher-risk drinkers based on drinking context
Source: PLOS Digit Health. 2024 Aug 21;3(8):e0000523. doi: 10.1371/journal.pdig.0000523 (PMC11338454; doi:10.1371/journal.pdig.0000523)
Supplement: S3 Appendix — (DOCX) [file pdig.0000523.s003.docx]

**S3 Appendix:** Qualitative codebook

| Name | Description |
| --- | --- |
| Adult soft drinks | Dissatisfaction with existing soft drinks, perceived as being sugary expensive (in pub settings) and unhealthy. Some suggestions of adult soft drinks which were perceived as being special or different, something you don’t always have which feels like a treat. |
| Alternate with soft drinks | Some liked the idea of alternating alcohol drinks with non-alcoholic drinks, soft drinks, or water. This was seen as a way of staying in social events for longer whilst reducing alcoholic drinks consumed. Some saw this as a preferential strategy to diluting alcoholic drinks (e.g. shandies) as they still got to enjoy their favourite drink. Some disliked this strategy as wanted to avoid soft drinks (as above) or felt volume of liquid would be a problem. |
| Avoiding friends who will pressure you | Mixed views some felt this was a helpful strategy, others felt it was too extreme and that they did not want to cut people out of their lives. |
| Being part of a group | Drinking alcohol means you are included in a group. In social pub contexts most people did not like strategies that marked them out as being different from the group. Stealth measures (such as diluting drinks or presenting as a drinker) or no-lo’s allowed them to maintain membership of the group. |
| Being perceived as judgemental | By not drinking alcohol, worry they are perceived as being judgemental of those drinking. |
| Birthdays | Special occasion where expectations to drink are heightened. |
| Cheap alcohol at home | Financial strategies less popular in a home context as perception is drinking alcohol at home is cheaper so already saving money. |
| Christmas | Special occasion where expectations and opportunities to drink are heightened. |
| Concentrating on company | This can happen naturally but as a strategy not popular, perceived as being too calculated and ineffective. |
| Concerns others will think they have a 'problem' | Hesitation about asking for social support in reduction goals in case they are perceived to have a problem or not be in control of drinking. |
| Doing something else | Alternative activities to contexts which are more tied to alcohol. |
| Drink as a 'treat' | Drink perceived as being a treat or reward |
| Drink slower | Perceived as being less effective in a group when drinking tends to synchronise (relates to rounds). |
| Drink type | Preferred drink type can impact on strategies which work |
| Drinking after going out | Practice of drinking at home after going out |
| Drinking fewer drinks | Includes different strategies such as diluting or alternating which leads to a reduction in drinks consumed |
| Drinking for confidence | Some people drink before they go out to put them at ease or reduce anxiety. |
| Drinking for fun | Some people drink for fun, particularly in social settings. |
| Drinking to unwind | Some people drink as they believe it helps them to unwind. |
| Driving | Driving rather than drinking (relevant to out of own home only) |
| Familiarity | Participants tended to pick out things they had tried before. |
| Hangovers | Hangovers are discussed as a deterrent to drinking most of the time, sometimes it is ‘worth’ the hangover. |
| Holidays | Special occasion where expectations and opportunities to drink are heightened. |
| Leave early to avoid pressure | Unpopular as a strategy, people wanted to stay in social settings but have strategies to drink less. |
| Lower strength | Used to refer to drinking lower strength options within-categories (e.g. having a lower strength beer) but also switching between categories. Seemed to relate strongly to drink type. |
| Making plans in advance | Benefit of making decisions before start drinking. Applied differently in different contexts – related to not buying alcohol or only buying alcohol for that day in home context, in out of home contexts more relevant to different activities and expectation setting. |
| New information about drinking patterns | Highlighting importance of understanding drinking patterns and targeting contexts |
| New habits | Focused on making new habits to reduce drinking. |
| No and low alcohol (No-Lo) | Some felt no-los had improved significantly recently and could be a way of maintaining group belonging in pub settings or relaxation at home thereby not missing out. Others were not very keen on flavour, this related to preferred drink type. |
| Not buying alcohol | Highlighted as a way of reducing home drinking by making alcohol less accessible. Many felt they wouldn’t go out specifically for alcohol if they didn’t have it at home. |
| Not wanting to miss out | Preferred strategies which enabled them to not miss out on perceived social benefits of drinking or on perceived relaxing effects. |
| Old Habits | Old habits which need to be broken to achieve goal of alcohol reduction. |
| Only buy what you want that day | Another way of reducing accessibility to reduce consumption at home. Relates to not buying alcohol to keep at home. |
| Out of sight | Another way of reducing accessibility alongside the temptation to drink at home. |
| People want the fun version of you | Relates to expectations to drink alcohol at special events and occasions. |
| Pre-drinking | Highlighted to save money or a social practice, more amongst younger people. |
| Presenting as a drinker | Stealth measure of having drinks which look like alcoholic drinks. Popular in pub settings with bigger groups to avoid pressure to drink. Relates to drink type. |
| Pressure to drink | Most participants had experienced pressure to drink particularly in social contexts. More common when people were younger. Relates to expectation to drink which is more implicit pressure. |
| Private alcohol reduction goals | Some participants were not comfortable seeking social support with alcohol reduction, felt it was private. Likely relates to being perceived as having a ‘problem’. |
| Problems with friendships | Highlighted as a downside of seeking social support. |
| Prohibitive cost of no-los | High price of many no-lo drinks highlighted as a barrier. |
| Ritual of drinking | Some participants highlighted the ritual nature of drinking practices. |
| Rounds | Rounds were seen as being a bit of an institution and difficult to avoid. Rounds with bigger groups were highlighted as a barrier to drinking less. |
| Set days for pub | A strategy to reduce alcohol consumption for a couple of participants was to only visit the pub on set days each week. |
| Setting expectations | Most participants felt that setting expectations that they would not be drinking or would be drinking a limited amount in advance of meeting friends would be helpful to offset pressure to drink. Some preferred not to highlight this as they felt this would increase pressure. |
| Smaller measures | More popular as a strategy in a home context, people felt they over-served when free pouring. Relates to drink type, more popular with spirits drinkers. |
| Smaller servings | Some people liked this as a strategy, but there were issues in terms of availability of smaller bottles (related to drink type) and value for money. |
| Social and physical setting | Participants highlighted different contextual elements that served as barriers to strategies to reduce consumption. |
| Social expectations to drink | Idea that drinking alcohol is the default option and that not drinking is different. Implicit pressure to drink. |
| Social support in sticking to goals | Some felt this would be helpful, others did not feel comfortable asking for support. |
| Socially acceptable excuses to abstain | Participants discussed socially acceptable reasons not to drink alcohol they used to avoid pressure to drink or to decrease defensiveness from those drinking. Relates to social expectations to drink and pressure to drink. |
| Soft drink at same time | Some thought this was a good strategy to slow drinking. Most preferred water over soft drinks. |
| Spend money on other things | Strategy of choosing to spend money spent on alcohol on a treat. Mixed views, highlighted need to keep track of money ‘not spent’ on alcohol. |
| Spending less | Spending less was more motivating in contexts outside the home, alcohol bought for home consumption was perceived as being cheaper and therefore already cost saving. |
| Support from partner | Most felt this would be helpful if done in an informal way. |
| Time limits | People liked the idea of time limits as a relatively simple rule to follow that could be decided in advance. Some liked the idea of starting drinking later (more at home) and having a cutoff point was more popular when out as it allowed you to cut out the drinking later in the night when less inhibited. |
| Value for money | Presented as a barrier to some of the strategies, particularly around strategies for at home-drinking focused on smaller servings or avoiding multipacks. |
| Watering down drinks | Another strategy on making drinks last longer to reduce overall drinking, relates to shandies, spritzers etc. Some people preferred doing this separately by alternating as they felt dilution would spoil their enjoyment. |
| Weekends | Highlighted as a time when drinking occasions would be heavier. |
| Will power | Highlighted as underpinning success of many of the strategies to reduce consumption. |
