# Supplementary material for: Cutting consumption without diluting the experience: Preferences for different tactics for reducing alcohol consumption among increasing-and-higher-risk drinkers based on drinking context
Source: PLOS Digit Health. 2024 Aug 21;3(8):e0000523. doi: 10.1371/journal.pdig.0000523 (PMC11338454; doi:10.1371/journal.pdig.0000523)
Supplement: Attachment — Submitted filename: Response to Reviewer comments.docx [file pdig.0000523.s004.docx]

**Response to Reviewer comments**

**Reviewer #1**

Competent research study of public health and substance use reduction interest, highlighting the importance of accounting for drinking practices and offering tailored support within alcohol reduction interventions.

We thank the reviewer for their positive comments.

**Reviewer #2**

I've thoroughly reviewed your paper and offered constructive feedback to enhance its impact. Kindly consider my suggestions to improve clarity and effectiveness. Excited to see the updated version. Best wishes.

We thank the reviewer for their time in reviewing our paper and their really helpful suggestions which we have incorporated as described below.

Abstract

1. instead of saying "Some contexts are likely more modifiable than others," consider rephrasing to "Certain drinking contexts may be more amenable to change than others."

We have adopted this wording here and in the introduction.

2. Provide a brief overview of the methodology used in the study, such as the specific techniques employed in developing context-specific strategies and the rationale behind using focus groups for data collection. This would help readers understand the research approach at a glance.

We have added some detail on the development of the strategies/tactics:

“Using the Behaviour Change Technique Taxonomy, context-specific tactics to reduce alcohol consumption were developed by the research team and revised following consultation with experts in behaviour change.”

The rationale for using focus groups, we have included in the manuscript as we are already at the abstract word limit:

“If focus groups run as intended, a conversational dynamic is established between participants. This facilitates discussion of broad opinions, attitudes, and past experiences (27,28). This process can lead to participants asking questions and exploring topics and ideas that a researcher in a one-to-one interview may not have broached (28). Here, the aim of the focus groups was to explore a range of opinions reflecting the experiences of a diverse group of increasing-and-higher-risk drinkers who drink in a range of drinking contexts.”

Introduction

1. explicitly state the gap in existing research that this study aims to address. For example, you could emphasize the lack of interventions tailored to individuals' drinking contexts and the potential effectiveness of such tailored approaches.

This has been strengthened in paragraph 3 of the introduction:

“Most existing alcohol reduction interventions do not account for variability in drinking practices. Instead, interventions tend to focus on reducing alcohol consumed without attending to context. However, previous research suggests drinkers do not conceptualise their alcohol consumption in terms of a weekly total, but rather as individual drinking occasions that are differentially integrated, important and acceptable in drinkers’ daily lives(17–19). Tailoring intervention strategies to individuals’ drinking contexts, and particularly those contexts in which individuals drink to harmful levels, may be more effective than a ‘one-size-fits-all’ approach. As such, studies exploring context-specific strategies for alcohol reduction are of value.”

2. When discussing the variability of drinking contexts and the importance of viewing alcohol consumption as a social practice, consider providing specific examples to illustrate these points. For instance, you could describe typical drinking occasions in different contexts, such as social gatherings with friends or relaxing evenings at home.

This has been included in paragraph 1:

“In the UK, the contexts in which people drink (e.g. socialising in the pub with friends or drinking at home with a partner) are highly variable(8–10).”

3. why such interventions are particularly relevant and effective for addressing alcohol reduction. Highlighting the accessibility, scalability, and potential for personalization of digital interventions would strengthen this argument.

This has been strengthened in paragraph 4:

“Digital interventions, such as software applications (‘apps’), offer substantial potential for delivering personalised intervention strategies, while addressing barriers associated with face-to-face interventions and reaching a significant proportion of the population (20).”

4. Provide a brief explanation of why the Behaviour Change Technique (BCT) Taxonomy was chosen as the framework for developing context-specific intervention messaging. Discuss how this taxonomy helps identify effective behaviour change techniques and tailor interventions to specific contexts.

This has been strengthened in paragraph 4:

“For the present study, the research team used the Behaviour Change Technique (BCT) Taxonomy(24,25) to develop context-specific intervention messaging for two of the existing Drink Less components; Insights and Action Planning. The BCT Taxonomy offers a reliable, cross domain, method for specifying, interpreting and implementing the active ingredients of behaviour change interventions (24). For example, within the Action Planning component, the BCT “facilitate goal setting”(24) could be differentially applied to particular drinking contexts. Specifically, someone who consumes most of their alcohol in the pub with friends could be prompted to alternate alcoholic drinks with soft drinks.”

5. Where relevant, connect the current study to previous research on alcohol reduction interventions, digital health interventions, and the effectiveness of context-specific approaches. This would help situate the study within the existing literature and highlight its novelty and contribution to the field.

There is currently no evidence we are aware of that evaluates the efficacy of context-specific tactics for alcohol reduction, this is now highlighted as the gap we are addressing and is highlighted as a priority for future research in the discussion. We do discuss relevant prior research on the work done to date highlighting the need to look at alcohol consumption as an event level practice (paragraph 2) and previous research on alcohol and digital interventions (paragraph 3 and 4) which has been expanded upon in response to comments from all reviewers.

6. Clearly articulate what makes this study novel and innovative compared to previous research. This could include its focus on context-specific interventions, the use of the BCT Taxonomy for intervention development, or its exploration of increasing-and-higher-risk drinkers' perspectives on alcohol reduction strategies.

This has been added to the final paragraph of the introduction:

“We are aware of no research to date which has examined increasing-and-higher-risk drinkers’ views on the applicability of strategies for alcohol reduction tailored to different drinking contexts. This study used a focus group design to examine these views.”

Method

1. Provide more details about the purposive sampling strategy used to select participants. Explain how participants were recruited from the existing database and the rationale behind selecting individuals with different ages, genders, and socioeconomic positions.

This has been added:

“Participants were recruited from an existing database made up of people who have previously taken part in alcohol reduction or smoking cessation studies and given permission for the research team to recontact them about research studies. Participants were emailed with study information and a link to the screening survey. Participants were also recruited via physical posters around the University campus which featured a QR link to the screening study and digital advertisements on social media accompanied by a link to the screening survey.”

“To ensure the inclusion of diverse viewpoints, the study used a purposive sampling strategy to ensure a maximum variation sample, with representation of different ages and genders, and we aimed to recruit at least half the sample from more disadvantaged socioeconomic positions (SEP).”

2. Expand on the rationale for choosing focus groups as the primary method for data collection. Discuss why focus groups were considered suitable for exploring participants' perspectives on alcohol reduction strategies in different contexts, particularly among increasing-and-higher-risk drinkers.

This has been added:

“If focus groups run as intended, a conversational dynamic is established between participants. This facilitates discussion of broad opinions, attitudes, and past experiences^23,26^. This process can lead to participants asking questions and exploring topics and ideas that a researcher in a one-to-one interview may not have broached^24^. Here, the aim of the focus groups was to explore a range of opinions reflecting the experiences of a diverse group of increasing-and-higher-risk drinkers who drink in a range of drinking contexts.”

3. Offer additional details about the data collection process, including how focus groups were conducted, how facilitators managed group dynamics, and any strategies used to ensure participants felt comfortable sharing their opinions.

We have added detail to the procedure section giving more detail on the focus groups and on facilitating group dynamics:

“After an icebreaker, there was a short presentation on the Drink Less app, the two relevant components and the plans for the context-specific updates. Participants then discussed how they would feel about receiving feedback on the types of drinking contexts they tended to be in when they did not achieve their goals. Then participants jointly completed a ranking task for two different drinking contexts, putting the alcohol reduction strategies developed by the research team for each context in order of least to most helpful, the aim of this task was to stimulate discussion of each strategy. Throughout the focus groups, the facilitators attempted to ensure that everyone shared views and attempted to draw out differences in opinion by asking whether any participants saw things in a different way. One example of this is following the group ranking task, participants were asked to select the strategies they thought would be personally more or less helpful, this was to draw out differences in opinion within groups. See Appendix 1 for the full topic guide.”

The point on participants feeling comfortable to share views is included in the supporting information:

“We aimed to create a non-judgemental and safe environment in which participants felt able to express their views honestly and openly. To achieve this, the lead facilitator MO shared that they drink alcohol, sometimes more than they intend to. Participants were also told that they could share views in the third person if they preferred, so rather than talking about what they might find helpful, focusing on ‘what could be helpful for other people’.”

4. Provide a clearer explanation of how context-specific intervention messaging was developed for the Drink Less app. Discuss the theoretical frameworks and behavior change techniques used to inform the development of these messages and how they were tailored to different drinking contexts.

We have added detail on the development process:

“ Next, the research team developed context-specific intervention messaging for two existing components of the Drink Less app, i) Insights and ii) Action Planning. This drew on theories (e.g. COM-B(34)) and the Behaviour Change Techniques (BCT) taxonomy(24).

The Insights component gives users weekly feedback on their progress towards meeting their goals. The research team developed messaging which could be delivered within the Insights component. This highlighted the types of contexts individuals were drinking in when they did not meet their goals (e.g. when you drink more than you want to, you tend to be in occasion X).

Within the Action Planning component, users make action plans to facilitate them reaching their goals. These take the form of implementation intentions, or “If... Then...” plans,(35) and can be differentially applied to particular drinking contexts (e.g. someone who consumes most of their alcohol at home alone could be prompted to not buy alcohol to keep at home or buy smaller bottles of alcohol). The team therefore developed suggested action plans (described as alcohol reduction strategies throughout) that the app might prompt the user with, which were specific to eight different drinking contexts(33): Alone at home, With partner or family at home, Social event in a home, Pub with friends, Pub alone, Big day or night out, Meal out and Out with Partner. There was some overlap in the strategies between different contexts (e.g. “I will only buy the alcohol I want to drink that day” was relevant for Alone at home and With partner or family at home). These action plans were developed with reference to the BCT taxonomy. For example, the BCT “facilitate goal setting” could be differentially applied to drinking contexts. Someone who consumes alcohol at home alone could be prompted to set goals such as to ‘use a measure when pouring spirits or wine’ or ‘buy smaller package sizes in the supermarket’. Alternatively, an individual more likely to consume multiple drinks in pubs with friends may set goals ‘to order soft drinks between alcoholic drinks’.

MO initially developed suggested intervention content for both components, this was then extensively reviewed and edited by the full research team, and wider experts in behaviour change and intervention development in a workshop.”

5. Provide a more detailed explanation of the data analysis approach, particularly the constant comparison analysis method. Describe how the coding process was conducted, including the steps involved in open coding, axial coding, and selective coding.

Have expanded on description of constant comparison method. Each stage of coding is described and this maps on to table two where you can see the output of each stage of coding:

“Transcriptions were pseudo-anonymised [gender, age, focus group number], where there were duplicates a, b were added after age. Constant comparison analysis(27,36) of transcripts was then undertaken. Constant comparison enables consecutive analysis of focus groups, to establish whether codes and themes present in earlier groups are seen in later groups.

This involved three stages of coding(36), see Table 2 below which maps each stage of coding;

1. Open coding – transcripts were read multiple times and codes were attached to chunks of text summarising the topic being discussed.
2. Axial coding – these codes were then grouped into categories with other codes that expressed similar or related topics.
3. Selective coding - themes were developed that expressed the content of the categories.
6. explain how themes and broader approaches were derived from the data and how the analysis incorporated both deductive and inductive coding approaches.

More detail on this has been added:

“The analysis approach was a mix of deductive and thematic coding. The intervention content developed by the research team framed much of the discussion in the focus groups and therefore many of the open codes developed were deductively coded in relation to this. However, participants were also encouraged to discuss tactics they felt were missing or had not been included and new tactics raised by participants were inductively coded. Themes were inductively coded from the data; they were driven by the way participants grouped and talked about different tactics and the factors that were perceived as influencing the applicability of tactics to different settings. When interpreting the categories and formulating themes, the notes taken during focus groups, those documenting conversations directly after focus groups and notes taken during coding were reviewed and this helped inform the structure of the themes.”

7. Provide a clearer discussion of data saturation and how it was assessed in the study. Explain how theoretical saturation was reached, particularly given the smaller sample size than originally planned.

We have expanded on this:

“While we recruited fewer participants than originally planned (n=20 rather than the estimated n=24, though the same number of focus groups was conducted), when analysing data from the last focus group, no new open codes were developed nor did the final focus group change the meaning of any existing codes or themes. As such and in line with previous definitions (27), the research team concluded that theoretical and meaning saturation had been achieved.”

Results

•	Provide more context about the screening survey and eligibility criteria to better understand the participant selection process.

We have not included additional detail on these topics, as we are not sure which further information the reviewer is requesting.

The eligibility criteria are outlined in the participant’s section:

Eligibility was determined via a screening survey. To be eligible for participation, participants had to be increasing-and-higher-risk drinkers (scoring ≥5 on the AUDIT-C(30)) and interested in using an alcohol reduction app now or in the future.

And the screening survey is described in the procedure section:

“Interested participants consented to the study and completed a screening survey including questions on alcohol consumption (AUDIT-C(30)), willingness to use an alcohol reduction app now or in the future and types of occasions participants typically drank in. Eligible participants were invited to one of four 90-minute focus groups.”

If the editor or reviewer feels any additional specific detail is required, we are very happy to provide it.

Discussion

•	While the four broad approaches are mentioned, provide concise definitions or examples for each approach. This ensures readers have a clear understanding of what each strategy entails. For instance, expand on what "Setting new habits" entails—does it involve replacing drinking routines with healthier alternatives, or incorporating non-drinking activities into social events?

We have added in details for each broad approach:

“These broad approaches encompassed the alcohol reduction strategies, and were: Diluting and substituting drinks (e.g. through lower strength drinks, smaller drinks or no-lo drinks), Reducing external pressure to drink (e.g. by reducing expectations around drinking or asking for support from friends), Creating barriers to drinking (e.g. by avoiding having alcohol in the house or by setting time limits on drinking) and Setting new habits (e.g. breaking old patterns and taking up new hobbies).”

•	While the text references previous research on drinking motives and situational pressures, integrate these findings more seamlessly into the discussion.

We think that the points we are making here are clearly expressed so are unsure how to meet this suggestion. If the editor or reviewer has a specific suggestion for how this could be more seamlessly communicated, we are very open to it.

•	Rather than simply mentioning parallels with established questionnaires, discuss how these findings contribute to a deeper understanding of participants' behaviours and preferences.

We have expanded on this:

“The Drinking motives raised in this study draw parallels with established drinking motives questionnaires(40). Participants discussed drinking to have fun which mapped on to both ‘social motives’ and ‘enhancement motives’. There was also the drinking motive to belong, which mapped well onto the ‘conformity’ motivation. Coping motivations were mentioned less frequently. Participants did talk about drinking to relax and for confidence, and three participants mentioned this in the context of feeling stressed, socially anxious or nervous in networking contexts. Previous qualitative research has found that participants tend to blur the line between drinking to relax and drinking to cope(41). As such, this study may broaden our understanding of how people think and talk about their own drinking. It could be that drinking to relax feels more palatable than drinking to cope, or that coping motivations may be perceived by others as less socially acceptable and might be indicative that they are not in control of their drinking.”

•	offer potential solutions or avenues for future research to address these concerns, such as exploring alternative pricing strategies for no-lo drinks or implementing targeted recruitment methods to include diverse participant demographics.

We do talk about pricing policies for no lo drinks:

“These findings support the need for pricing policy changes which ensure price differentials between no-lo and standard alcohol drinks and remove pricing structures that disincentivise smaller purchases.”

We have added some ideas for more targeted recruitment options:

“Whilst the aim of focus groups is not to be representative it is important to include diverse voices in intervention development research to ensure person-focused and fit-for-purpose interventions, as such alternative methods or recruitment (e.g. by leafleting in less advantaged areas) or data collection (e.g. offering alternative formats or holding focus groups or interviews in more convenient locations) could be explored in future studies.”

•	provide recommendations for app developers, policymakers, or public health practitioners based on the study's insights would enhance the conclusion's relevance and applicability.

We do offer some implications throughout (example below) but given the efficacy of the individual strategies have not yet been examined we think it is important not to overstate the studies implications:

“These findings support the need for pricing policy changes which ensure price differentials between no-lo and standard alcohol drinks and remove pricing structures that disincentivise smaller purchases.”

We also added:

“This study centres user voices and highlights broad approaches to alcohol reduction which were deemed as appropriate for different drinking contexts. However, the developed strategies have not undergone efficacy testing, this is now a priority for future research.”

**Reviewer #3**

This study on the context-dependency of strategies for reducing alcohol consumption is highly interesting and much needed. There are some issues with the manuscript that need to be addressed before this work can be considered publishable, though. The qualitative perspectives should have more voice in the study of such a complex phenomenon as alcohol consumption. I also have a major concern with the conceptional framing of the manuscript, as well as some minor suggestions for improvements.

We thank the reviewer for their time, for their positive comments and their helpful suggestions which have strengthened the paper.

The major issue is the application of the concept “strategies” in the context of social practices. Here, I can see some theoretical misalignments. Even in the table of thematic coding, the strategies are quite central. Is it appropriate to call it strategies when the final act of the social practice of alcohol consumption should be on the individual level? Would that not rather be a tactic? In a nutshell, strategies are top-down and initiated by those governing populations to governmentalize their behaviour while tactics are bottom-up and employed situationally by individuals to navigate social situations. As an example of this, consider how this distinction is employed in work on patient empowerment:

https://eur01.safelinks.protection.outlook.com/?url=https%3A%2F%2Fdoi.org%2F10.1177%2F1363459319831343&data=05%7C02%7Cm.oldham%40ucl.ac.uk%7Cc63d3d07a0f54e1ee08708dc847848d7%7C1faf88fea9984c5b93c9210a11d9a5c2%7C0%7C0%7C638530899361269709%7CUnknown%7CTWFpbGZsb3d8eyJWIjoiMC4wLjAwMDAiLCJQIjoiV2luMzIiLCJBTiI6Ik1haWwiLCJXVCI6Mn0%3D%7C0%7C%7C%7C&sdata=0AGZBO0BLKKx93yZDFYTOMjuHo5AOkMWqEkGJDtiHRY%3D&reserved=0

This is not to say that this manuscript needs to follow the same path, but it would be advisable to clearly position the use of the concept “strategies” (such as in “coping strategies” a la Lazarus, R. S., & Folkman, S. (1984). Stress, appraisal, and coping. Springer publishing company) or employ another term such as “tactics”.

We appreciate the reviewer’s thoughtful comment. We have looked at both suggested readings and agree with the reviewer that the ‘tactics’ terminology is more accurate than ‘strategies’. This has been replaced throughout.

Detailed suggestions for improvement and strengthening the contributions of the manuscript:

•	Please, reformulate line 88-89: Theories, including Social Practice Theory, highlight the importance of viewing alcohol consumption as an event, occasion or practice-level phenomenon (8,11). Both references are applying the theory and not necessarily presenting the one.

Good point, this has been reformulated:

“When conceptualising alcohol consumption, researchers have applied theories such as Social Practice Theory, to emphasise the importance of viewing alcohol consumption as an event, occasion or practice-level phenomenon (8.11).”

•	Lines 89-91 please, reformulate or provide references to this statement : “This requires researchers to move away from thinking about alcohol consumption as a single health behaviour and to instead think of alcohol consumption as one constituent part in a range of social practices.” Which researchers say this? How can alcohol consumption be a health behavior when research is every day more strongly agreeing on the negative effects and consequences of such? Maybe “a health- related behaviour”? Or “health-related practices with direct impact on the health outcomes”?

We see your point. We meant that reducing drinking to one single behaviour may be reductive as different drinking contexts hold different meanings and value for people, so therefore it is important to understand the wider social practices in which drinking occurs. However, we think this point is made more clearly in the next paragraph, so we have removed this sentence.

•	More reflections on the different contextualization of the methods applied are needed. The F2F focus groups are not the same as the technologically mediated ones. What limitations and differences are there?

This is a good point and we have expanded on this in the reflections section in the supporting information:

“The difference in modalities did seem to have some impact on the dynamics of the focus group. It seemed easier in person for participants to signal to others that they had something to say and so a space was left for their insights. This was a little clumsier online with participants occasionally starting to talk at the same time, this may have led participants to be less willing to share views. However, having online focus did seem to be more inclusive and captured voices that otherwise may not have been heard.”

•	Regarding the coding, can the three-stage approach be explained in some more detail? Please also add a discussion of how the themes raised during the focus groups that the facilitator uncovered were employed in the coding process?

We have expanded on this:

“Constant comparison analysis(27,36) of transcripts was then undertaken. Constant comparison enables consecutive analysis of focus groups, to establish whether codes and themes present in earlier groups are seen in later groups.

This involved three stages of coding(36), see Table 2 below which maps each stage of coding;

1. Open coding – transcripts were read multiple times and codes were attached to chunks of text summarising the topic being discussed.
2. Axial coding – these codes were then grouped into categories with other codes that expressed similar or related topics.
3. Selective coding - themes were developed that expressed the content of the categories.

The analysis approach was a mix of deductive and thematic coding. The intervention content developed by the research team framed much of the discussion in the focus groups and therefore many of the open codes developed were deductively coded in relation to this. However, participants were also encouraged to discuss tactics they felt were missing or had not been included and new tactics raised by participants were inductively coded. Themes were inductively coded from the data; they were driven by the way participants grouped and talked about different tactics and the factors that were perceived as influencing the applicability of tactics to different settings. When interpreting the categories and formulating themes, the notes taken during focus groups, those documenting conversations directly after focus groups and notes taken during coding were reviewed and this helped inform the structure of the themes.”

•	Females are overrepresented in both the sample and the data presentation. Please reflect on the reasons and implications. Also, how as “female” established? Self-reported or observed? How does it impact the results of the study? Such reflections should be integrated into limitations or discussion sections.

Gender was self-reported during the screening survey. The gender divide was 60% female so whilst there was some oversampling of female participants there was still a reasonable representation of male views so we don’t think this would have impacted on the results too much. We think that the weighting towards more advantaged participants is a much more serious limitation, so we currently have focused on that in the discussion. The paper is already quite long so with that in mind we would be interested in the editor or reviewers view of whether the 60/40 gender split should also be expanded on in the discussion.

**Reviewer #4**

Peer Review for: Cutting consumption without diluting the experience: preferences for different strategies for reducing alcohol consumption among increasing-and-higher-risk drinkers based on drinking context.

General Comments

I commend the authors for their valuable contribution to the field of alcohol intervention research. The use of focus groups to identify mechanisms that could aid in reducing alcohol consumption and integrating patient perspectives into the development of an intervention app is highly commendable and enriches the field. Including the patient voice in research not only increases the study's relevance but also enhances the potential for real-world applicability. Generally speaking, too little qualitative research that focuses on including the lived-experience of patients is still being conducted, and this article takes a good step into this direction.

We thank the reviewer for taking the time to review our work, for their positive feedback and their helpful suggestions which have strengthened the paper.

Strengths

Patient-Centered Approach: The inclusion of patient voices through focus groups is a significant strength. This approach ensures that the strategies identified are grounded in real-world experiences and needs, which is crucial for developing effective interventions.

Development Process: The methodical process of using qualitative data to inform the development of an intervention app is well-documented and provides a clear roadmap for future research in this area.

We thank the reviewer for their positive feedback.

Limitations

Lack of Efficacy Testing: A major limitation of the study is that the suggested strategies have not been tested for efficacy. This should be explicitly stated as it limits the research's usefulness for other researchers who may wish to build on this work. Without efficacy data, it is challenging to assess the potential impact of these strategies on reducing alcohol consumption.

This is a very good point! We have added this to the limitations section in the discussion:

“This study centres user voices and highlights broad approaches to alcohol reduction which were deemed as appropriate for different drinking contexts. However, the developed strategies have not undergone efficacy testing, this is now a priority for future research.”

Variation in AUDIT Scores: The focus groups appeared to differ in their Alcohol Use Disorders Identification Test (AUDIT) scores. It would be beneficial to conduct significance testing to determine whether these differences are statistically significant. If significant differences are found, presenting the outlier group separately could provide valuable insights into whether participants with particularly high AUDIT scores offer different suggestions.

We calculated 95% CIs for mean AUDIT scores for each of the four groups separately. They are group 1(6.40, 8.80), group 2 (8.00, 11.50), group 3 (6.50, 9.33), and group 4 (5.60, 6.80). Although mean AUDIT scores were higher in group 2 and lower in group 4, there is considerable overlap between the 95% CIs for each of the four groups. We have compared the transcripts and coding from groups 2 and 4 but there doesn’t seem to be any evidence of divergence in endorsement of different tactics. As such, we do not think it would be appropriate to formally analyse the transcripts separately.

Specific Recommendations

Explicit Limitation on Efficacy: Clearly state the limitation regarding the lack of efficacy testing in the discussion section. This transparency will help other researchers understand the scope and applicability of your findings.

This is a very good point. Included in the discussion:

“This study centres user voices and highlights broad approaches to alcohol reduction which were deemed as appropriate for different drinking contexts. However, the developed tactics have not undergone efficacy testing, this is now a priority for future research.”

Significance Testing for AUDIT Scores: Perform statistical analyses to determine if the focus groups differ significantly in their AUDIT scores. If significant differences are present, consider discussing the implications of these differences and presenting the data from the outlier group separately. This additional analysis could uncover nuanced insights and enhance the depth of your findings.

We explain this fully above but given the overlap in 95% CIs for the four groups we think it is more appropriate to present findings unstratified by group.

Overall, I recommend this article for publication, subject to the authors addressing the noted limitations and considering the suggested analyses. The study makes a valuable contribution to the field, and with these enhancements, it can offer even greater utility to researchers and practitioners working on alcohol intervention strategies.

Thank you for your thoughtful feedback, it has made the paper stronger.
